# Photon-counting CT demonstration of acute labyrinthine ischemia in idiopathic sudden sensorineural hearing loss: a case report

**DOI:** 10.3389/fmed.2025.1709516

**Published:** 2025-11-14

**Authors:** Xiujuan Sun, Jia Chen, Jingjing Xu

**Affiliations:** 1 Department of Radiology, The Second Affiliated Hospital, Zhejiang University School of Medicine, Hangzhou, China; 2 Department of Otolaryngology, The Second Affiliated Hospital, Zhejiang University School of Medicine, Hangzhou, China

**Keywords:** photon-counting CT, imaging, idiopathic sudden sensorineural hearing loss, ischemia, anterior inferior cerebellar artery (AICA)

## Abstract

The pathophysiology of idiopathic sudden sensorineural hearing loss (ISSNHL) remains unclear. It is widely hypothesized that vascular ischemia, leading to acute labyrinthine ischemia, may represent the most probable underlying etiology. We describe a 51-year-old previously healthy man presenting with acute-onset left-sided hearing loss, tinnitus, and vertigo persisting for 24 h. His clinical features were consistent with acute labyrinthine ischemia, suggesting this as the probable etiology of ISSNHL. Initial photon-counting CT (PCCT) of the inner ear suggested that the branches of the anterior inferior cerebellar artery (AICA) supplying the inner ear, including the labyrinthine artery, were slightly attenuated and exhibited a sparse branching pattern. Follow-up imaging after therapy within a week revealed significant improvement in both the vascular caliber and blood flow. To our knowledge, this case represents the first successful application of PCCT to directly suggest acute labyrinthine ischemia, providing the first reported radiological evidence for this mechanism.

## 1 Introduction

The pathogenesis of idiopathic sudden sensorineural hearing loss (ISSNHL) remains unclear, with vascular ischemia being widely considered a probable underlying etiology. The labyrinthine artery is the exclusive terminal artery supplying the inner ear, functioning as an end-artery without collateral circulation (1). Although labyrinthine artery pathology has been hypothesized to contribute to acute labyrinthine ischemia, direct visualization of this vascular structure has remained challenging with conventional imaging techniques. Compared to traditional energy-integrating detector CT (EID-CT), photon-counting CT (PCCT) offers superior spatial and contrast resolution, enabling more detailed visualization of anatomical structures. The visualization of inner ear anatomy is significantly enhanced by the advantages of PCCT. PCCT could be a valuable tool for diagnosing vascular-induced acute labyrinthine ischemia.

## 2 Case report

**Figure 1:**
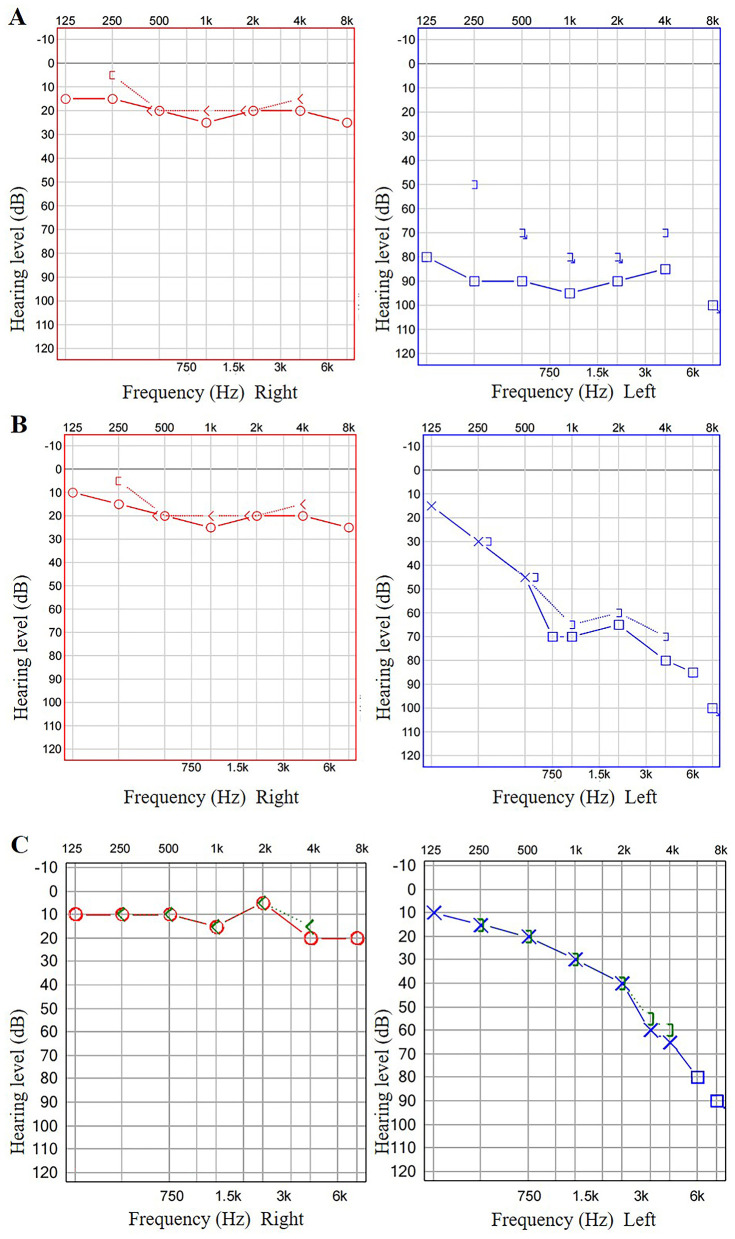
The patient's PTA results. The patient's PTA revealed severe sensorineural hearing loss across all frequencies in the left ear **(A)**. After 8 and 13 days, follow-up PTA suggested significant improvement in hearing thresholds across all frequencies in the left ear (**B**, **C**).

**Figure 2:**
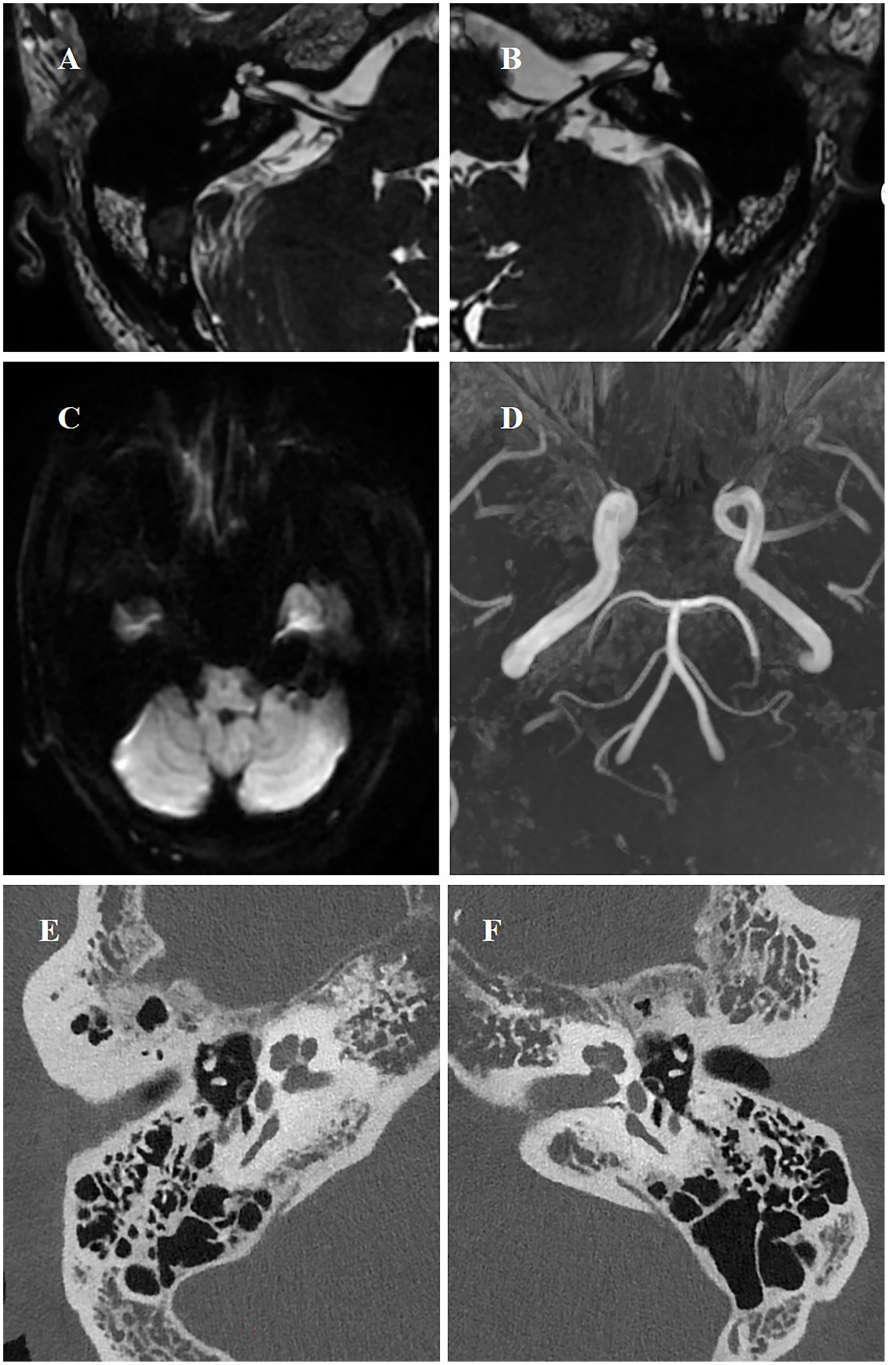
MRI, DWI, and PCCT at first presentation. Axial T2-weighted image **(A**, **B)** and DWI **(C)** showed no evidence of labyrinthine anomalies. MRI-3D TOF imaging showed no evidence of posterior circulation occlusion **(D)**. The inner ear unenhanced PCCT revealed that the osseous labyrinth and surrounding structures exhibited no significant abnormalities in morphology or density **(E**, **F)**.

A 51-year-old previously healthy man presented with acute left-sided hearing loss, tinnitus, and vertigo of 1-day duration. However, he denied any significant nausea, vomiting, ear pain, otorrhea, headache, or neck pain. His medical history was unremarkable. Normal external auditory canals and tympanic membranes bilaterally. CN VIII: Profound unilateral sensorineural hearing loss on the left (confirmed by tuning fork test). CN II-XII: Otherwise entirely intact, with specific note of normal facial nerve (CN VII) symmetry and function. Vestibular: no spontaneous nystagmus. Neurological: The remainder of the motor, sensory, and cerebellar examinations was non-focal. Pure-tone audiometry (PTA) showed severe sensorineural hearing loss across all frequencies in the left ear (Figure 1A). Vestibular testing revealed left horizontal semicircular canal dysfunction (asymmetry ratio: 0.36) and absent left vestibular evoked myogenic potentials (VEMPs). Otoacoustic emissions (OAEs) were bilaterally absent. Initial MRI, DWI, 3D-TOF were unremarkable (Figures 2A–D).

**Figure 3:**
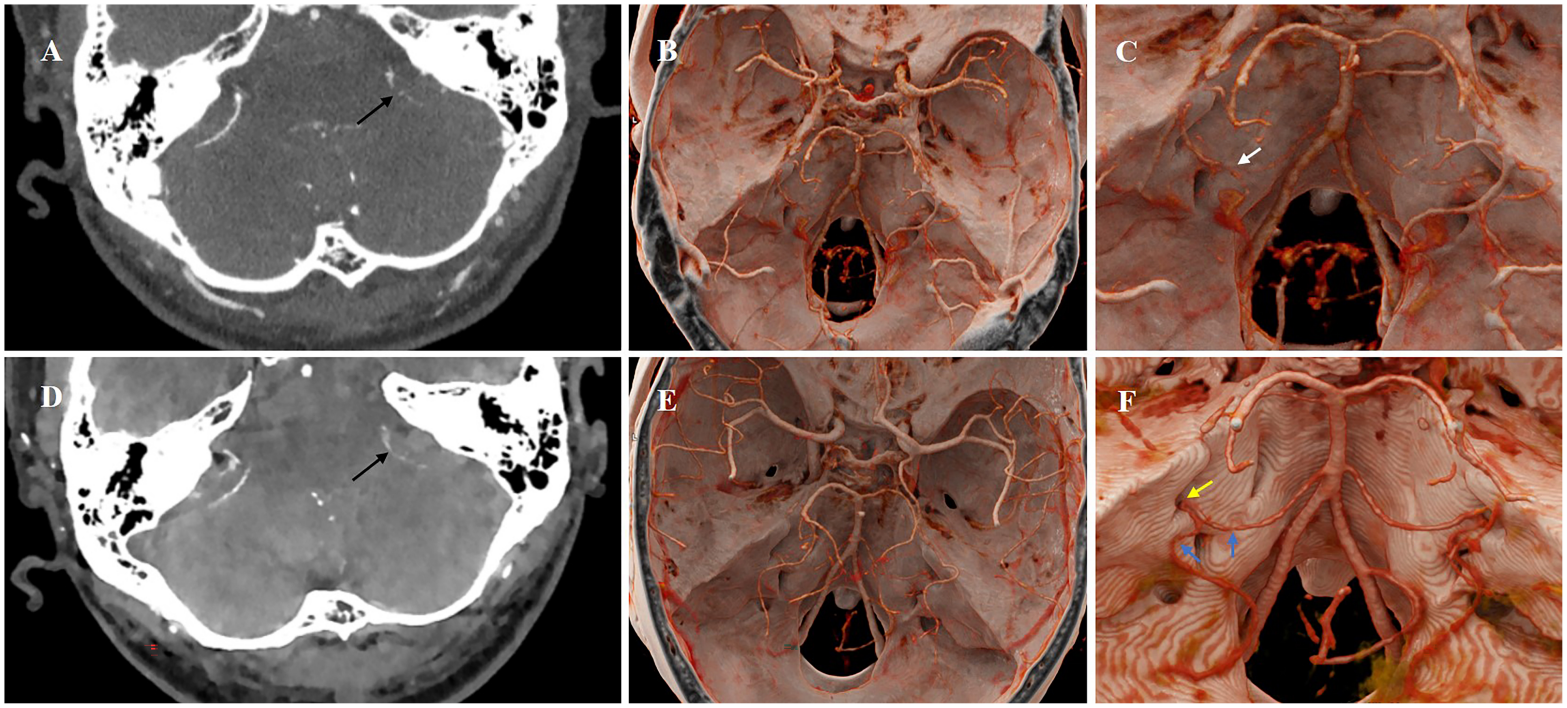
Pre- and post-treatment CTA with PCCT images of the inner ear. The inner ear CTA initially suggested that the left branches of AICA supplying the inner ear, including the labyrinthine artery, were slightly attenuated with sparse branching patterns **(A)**. Furthermore, the luminal visualization of some branch arteries was incomplete (**A**, black arrow) (**C**, white arrow), and the distal segments of some branch vessels were not visualized, suggestive of stenosis or occlusion. Pre-treatment 3D-reconstructed images **(B)** showed inferior vascular delineation compared to post-treatment **(E)**. After 12 days, the follow-up CTA revealed significant improvement in the caliber and blood flow of the branch arteries (**D**, black arrow). The 3D-reconstructed images **(F)** suggested that the left AICA and its branches still exhibited localized luminal stenosis (blue arrow), while the labyrinthine artery was already patent (yellow arrow).

The patient underwent a series of PCCT (NAEOTOM Alpha, Siemens Healthineers, Germany) examinations. Scanning parameters were as follows: 140 kV tube voltage, 144 × 0.4 mm collimation, 0.5-s gantry rotation time, pitch of 0.8, and tube current modulation (quality reference: 205 mAs). Images were reconstructed with a 1.0 mm slice thickness using a Qr40 kernel and iterative reconstruction at strength level 4. The inner ear unenhanced PCCT revealed that the osseous labyrinth and surrounding structures exhibited no significant abnormalities in morphology or density (Figures 2E, F). The inner ear computed tomographic angiography (CTA) with PCCT initially suggested that the branches of anterior inferior cerebellar artery (AICA) supplying the inner ear, including the labyrinthine artery, were slightly attenuated with sparse branching patterns (Figure 3A). Moreover, some of the branch arteries suggested incomplete luminal opacification, and the distal segments of some branch vessels were not visualized, indicative of potential stenosis or occlusion (Figures 3A, C). Pre-treatment 3D-reconstructed images (Figure 3B) showed inferior vascular delineation compared to post-treatment (within a week, Figure 3E). These imaging findings strongly support acute labyrinthine ischemia as the underlying pathological mechanism of ISSNHL.

The patient received combined therapy (ginkgo/betahistine, mecobalamine, steroids, batroxobin, retroauricular steroid injections, and hyperbaric oxygen). The patient's symptoms demonstrated marked improvement. Follow-up PTA at 8 and 13 days showed progressive hearing improvement—PTA improved from 90 dB to 65 dB at 8 days, and to 39 dB at 13 days (Figures 1B, C). After 12 days, the subsequent follow-up CTA with PCCT imaging revealed significant enhancement in both the vascular caliber and blood flow (Figure 3D). The 3D-reconstructed images (Figure 3F) suggested that the left AICA and its branches still exhibit localized luminal stenosis, while the labyrinthine artery was already patent. These imaging findings were most consistent with the clinical diagnosis. To the best of our knowledge, this is the first documented case of using PCCT to observe acute labyrinthine ischemia.

## 3 Discussion

PCCT represents the latest advancement in commercialized CT technology. Distinct from conventional EID-CT, PCCT employs semiconductor detectors that directly convert X-ray photons into electrical signals, eliminating the need for a scintillator layer. This direct conversion allows the detector to count individual photons and measure their energy, independent of energy-weighting. Furthermore, the absence of detector septa enables a smaller pixel size and superior spatial resolution. The effective removal of electronic noise also facilitates the use of ultra-low-dose protocols, positioning PCCT as a superior imaging modality.

Recently, PCCT has gradually been applied in clinical settings, representing an important advance in CT technology. PCCT offers better spatial resolution, higher contrast-to-noise ratio, elimination of electronic noise, improved dose efficiency, and routine multi-energy imaging (2). Rao et al. (3) reported that the visualization of inner ear anatomy is significantly enhanced by the advantages of PCCT. This improvement allows for better characterization of many structures, including the full extent of the vestibular and cochlear aqueducts, the cochlear cleft, and various nerves, as well as their anatomic relationships. To the best of our knowledge, only a few studies have investigated the use of PCCT for inner ear imaging thus far, and this is the first documented case of using PCCT to observe acute labyrinthine ischemia.

The labyrinthine artery is the sole terminal artery responsible for supplying blood to the inner ear. It originates from branches of the AICA and functions as an end artery, without collateral circulation. As it enters the inner ear, the labyrinthine artery divides into three main branches: the anterior vestibular artery (AVA), the main cochlear artery (MCA), and the vestibulo-cochlear artery (VCA). The VCA further bifurcates into two branches: the posterior vestibular artery and the cochlear ramus (1, 4–6). The patient's test results showed impairment of the left cochlea and vestibule, along with marked hearing loss, which points to inadequate perfusion by the labyrinthine artery resulting in left inner ear ischemia. The patient underwent a series of PCCT examinations, which provided excellent visualization of the inner ear anatomy. The inner ear CTA with PCCT initially suggested that the branches of AICA supplying the inner ear, including the labyrinthine artery, were slightly attenuated with sparse branching patterns. Furthermore, the luminal visualization of some branch arteries was incomplete, suggestive of stenosis or occlusion. Following therapeutic intervention, follow-up CTA revealed significant enhancement in both the vascular caliber and blood flow within the artery, the labyrinthine artery was already patent. Therefore, it is possible that the patient's labyrinthine artery stenosis leads to labyrinthine ischemia.

ISSNHL is clinically defined as a hearing impairment characterized by a decrease of more than 30 dB across three consecutive frequencies, occurring within a 72-h period, as measured by pure-tone audiometry. Epidemiological studies indicate that the annual incidence of ISSNHL ranges from 11 to 77 cases per 100,000 individuals (1, 7–11). Our patient presented with acute hearing loss in the left ear, accompanied by tinnitus and vertigo that had begun 1 day prior. These symptoms are consistent with this diagnosis. This acute and potentially disabling inner ear disorder typically manifests unilaterally, resulting in permanent hearing impairment that may range from mild to profound across the hearing spectrum, affecting both high and low frequencies. The condition is frequently accompanied by tinnitus and vertigo, significantly impacting patients' quality of life if not promptly diagnosed and effectively managed.

The pathophysiology of ISSNHL remains unclear, and most cases are idiopathic, which has been the subject of extensive debate (9). Various potential causes have been proposed, including vascular events, coagulation disorders, autoimmune diseases, inflammatory disorders and trauma (7, 12). Additionally, the potential pathogenesis of sudden deafness may involve vasoconstriction, vascular embolism or thrombosis, hair cell damage, and the accumulation of inner ear fluid. Since the inner ear requires a high-energy metabolism and the labyrinthine artery and its subdivisions are terminal arteries with minimal collateral circulation, the labyrinth is particularly susceptible to ischemia (4, 6, 13, 14). Even transient ischemia can result in permanent damage. Specifically, cochlear electrical activity deteriorates within 60 s of blood flow interruption and may fail to recover if the obstruction persists for more than 30 min (13). According to the literature, labyrinthine ischemia can be reflected through various imaging modalities. Occasionally, the labyrinth revealed a high-signal-intensity lesion on both fluid-attenuated inversion recovery (FLAIR) images (6) and DWI (15). After contrast enhancement and a 4-h delayed MRI scan of the inner ear, using 3D FLAIR sequences, a filling defect was identified within the posterior semicircular canal. Additionally, the signal intensities within the labyrinth were markedly elevated (9, 10). These findings were likely contributing to labyrinthine ischemia. However, none of them directly showed the condition of the labyrinthine artery. Notably, the application of PCCT in diagnosing labyrinthine ischemia has been scarcely documented in the literature. Therefore, it demonstrates that PCCT is a promising imaging modality for diagnosing the vascular etiology of ISSNHL.

As an advanced imaging technology, PCCT is increasingly being integrated into clinical practice as a routine diagnostic modality. While the initial capital investment exceeds that of conventional CT systems, the technology's fundamental advantage resides in its superior detector efficiency. This innovation enables substantial radiation dose reduction while maintaining diagnostic image quality, and simultaneously provides improved spatial resolution. These technical advancements—through enhanced diagnostic confidence and the potential to reduce additional imaging studies—are expected to redefine the long-term cost-benefit profile of this technology.

## 4 Conclusion

In conclusion, our findings suggest that PCCT imaging features provide valuable diagnostic information for evaluating labyrinthine ischemia. Furthermore, inner ear CTA in patients with ISSNHL reveals distinct vascular manifestations that may facilitate differential diagnosis and guide therapeutic decision-making. Nevertheless, the single-case design inherently limits the generalizability of our findings. Larger cohort studies with extended follow-up periods are necessary to validate the diagnostic utility and clinical significance of PCCT in patients with labyrinthine ischemia. This limitation underscores important directions for future research, which we plan to address through systematic investigations.

## Data Availability

The raw data supporting the conclusions of this article will be made available by the authors, without undue reservation.
